# Rural Practice Made Attractive: A Scoping Review of Rural Primary Care Physician Recruitment and Retention Incentives

**DOI:** 10.1007/s11606-026-10218-8

**Published:** 2026-01-26

**Authors:** Kelley Arredondo, Katherine Bay, Laura Witte, Hilary Touchett, Mandi Sonnenfeld, Alexander Paterson-Roberts, Matthew Vincenti, Bradley V. Watts

**Affiliations:** 1 Houston VA HSR&D Center for Innovations in Quality, Effectiveness, and Safety, Michael E. DeBakey VA Medical Center, Houston, TX USA; 2 Department of Medicine, Baylor College of Medicine, Houston, TX USA; 3 VHA Office of Rural Health’s Veterans Resource Center in White River Junction, White River Junction, VT USA; 4 VA South Central Mental Illness Research, Education, Clinical Center, a Virtual Center, Houston, TX USA; 5 Veterans Affairs, South Texas Veterans Health Care System, San Antonio, TX USA; 6 Department of Psychology, University of Houston, Houston, TX USA; 7 Department of Medicine, Dartmouth Geisel School of Medicine, Hanover, NH USA; 8 Department of Psychiatry, Larner School of Medicine at the University of Vermont, Burlington, VT USA

**Keywords:** rural workforce, primary care, rural health, retention, recruitment

## Abstract

**Background:**

Workforce development programs aim to address the disparity in the number of primary care physicians (PCPs) practicing in rural U.S. areas. Both publicly and privately funded rural recruitment and retention programs have worked for decades to enhance healthcare access. However, little is known about their comparative effectiveness in retaining PCPs long term. This scoping review assessed the success of programs in retaining rural PCPs.

**Methods:**

We searched PubMed, Embase, and Health Business Elite for peer-reviewed literature, published between 2013 and 2023, focusing on PCPs’ rural recruitment and retention in the U.S. The gray literature search included sources like the Rural Health Information Hub and the Rural Medical Training Collaborative, followed by a Google search. Articles were screened by two authors, with discrepancies resolved by a third.

**Results:**

From 2227 articles identified, only 10 met the inclusion criteria, with 7 additional programs found through gray literature, totaling 17 programs. Financial incentives, such as state loan repayment programs (*n* = 2) and scholarships (*n* = 2), showed the highest reported retention rates (50–100%) in rural areas; however, these results should be interpreted cautiously due to small sample sizes and substantial variability in follow-up periods across programs.

**Discussion:**

Most rural PCP workforce development programs did not report retention outcomes. Among those that did, financial incentive programs had higher retention rates than rural education and residency programs. These findings are limited by the heterogeneity of results reported and the variability in program sample sizes. Additionally, many education programs reported the number of clinicians in rural areas but did not specify whether they were in primary care. Future program reports and research should standardize reporting to include the number of individuals who completed the program, their specialty, duration of rural practice post service time commitment, and current practice status in rural areas.

**Graphical Abstract:**

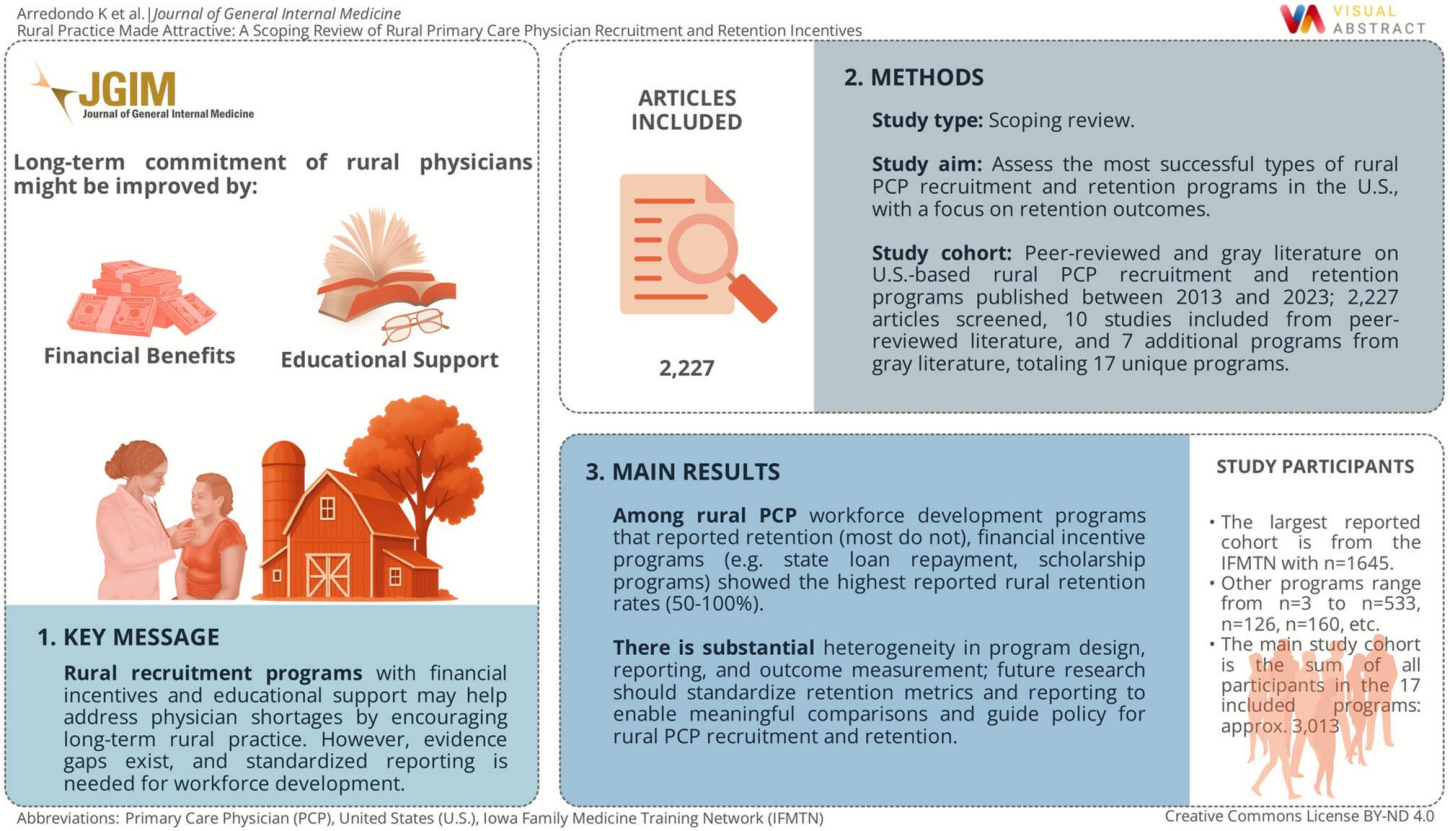

**Supplementary Information:**

The online version contains supplementary material available at 10.1007/s11606-026-10218-8.

## INTRODUCTION

Over 46 million United States (U.S.) residents live in rural areas;^1^ however, the ratio of primary care providers (PCPs) serving urban residents outnumbers the ratio serving rural residents by over 50%—at 8.0 PCPs in urban areas and only 5.1 in rural areas per 10,000 residents.^2,3^ To close this gap, publicly and privately funded recruitment and retention programs have sought to extend healthcare access to rural residents for decades.^4,5^ Yet, despite considerable investment in resources and programs, PCP shortages persist in rural areas within the U.S.^2,6^ While past research has identified several factors contributing to rural PCP shortages (e.g., less resources, less vacation time),^7^ less is known about the comparative effectiveness of rural PCP recruitment and retention programs for long-term PCP commitment to rural practice. Given the resources invested into these programs, it is paramount to evaluate and compare their success to allocate support for the most effective strategies.

Primary care is the foundation of the U.S. healthcare system.^8,9^ PCPs deliver accessible, continuous, and comprehensive care; reduce avoidable downstream utilization; and play a key role in chronic disease management and prevention.^10^ Americans living in rural areas consistently have less access to care and exhibit less engagement in preventive care than their urban counterparts, often increasing preventable disease and burden.^11–15^ Thus, rural PCPs face a unique set of recruitment and retention challenges, such as large and complex patient panels, greater professional isolation, heavier administrative burden, and fewer opportunities for career advancement compared to urban settings. These factors compound and make rural primary care positions particularly difficult to fill and sustain over time and underscore the need to evaluate rural PCP’s specific workforce programs rather than physician programs more broadly.

This review focuses on physicians (MD and DO), but we acknowledge that primary care in rural communities is also provided by nurse practitioners, physician assistants, and, in some areas, midwives. These clinicians are not included in the current scoping review because their scope of practice and role in primary care can vary significantly across states, which affects the comparability of workforce development programs targeting these groups.

Frequently, recruitment and retention program evaluations consist of reporting how many physicians have participated in the program,^16^ completed the program,^17^ or intend to practice in a rural area.^18,19^ Although a beneficial first step, this type of evaluation does not provide insight into how many PCPs continue practicing in rural areas after program completion. Previous reviews have also explored the effectiveness of rural recruitment and retention efforts for the general medical workforce^20^ or PCPs in international settings.^21^ However, a paucity of evidence exists demonstrating the comparative value of these programs for retaining PCPs in rural U.S.

The World Health Organization (WHO) recommends rural workforce development interventions include one or more of four main categories: education, regulation, financial incentives, and personal and professional support.^20,22^ Many existing U.S.-based rural PCP recruitment and retention programs align with these WHO recommendations. For example, regulatory programs include federally funded J-1 visa waivers that eliminate the requirement of J-1 visa holders to return home for 2 years once they complete training in return for 3 years of service in a health professions shortage area (HPSA).^23,24^ Moreover, research evaluating the efficacy of individual programs exists. Kahn et al. reported that physicians in Washington State who participated in J-1 visa waivers stayed an average of 23 months longer than the required service time commitment; however, after leaving their J-1 waiver employer, 74% (35 of 47) now practiced in urban areas.^25^ Similarly, researchers evaluating the efficacy of state loan repayment programs versus J-1 visa waivers in rural Nebraska found that those participating in state loan repayment programs were more likely to stay in rural Nebraska than physicians enrolled in J-1 visa waivers.^26^ Very few studies compare program efficacy, and, to our knowledge, little to no studies compare the characteristics and effectiveness specifically of PCP recruitment and retention programs across categories (i.e., education, regulation, financial incentives, and personal and professional support).

Scoping reviews are useful for identifying knowledge gaps, mapping out a body of literature, clarifying concepts, and examining research practices.^27^ Thus, this review aids in understanding whether and how program effectiveness is currently evaluated and seeks to identify which programs have been most successful. Evaluation of these programs is critical to identify program characteristics that enhance rural PCP retention.

The objective of this scoping review was to assess the PCP workforce (i.e., population) in relation to workforce development recruitment and retention programs (i.e., concept) in rural areas (i.e., context) as defined by the author(s). *Recruitment* programs are defined as those offered to physicians who are not currently practicing in rural areas, while *retention* programs are offered to physicians currently practicing in rural areas. Specifically, we sought to answer the following research questions:

1. What rural PCP recruitment and/or retention programs have been shown to increase rural healthcare provider retention?
2. Which programs implemented to overcome physician shortages in rural areas are most successful at retention?

## METHODS

### Protocol and Registration

The protocol for this study has been registered with the Open Science Framework (10.17605/OSF.IO/C26Y5).

This scoping review was conducted using the Arksey and O’Malley framework to systematically investigate literature addressing the primary and secondary review questions.^28^

This review follows the Preferred Reporting Items for Systematic Review and Meta-Analysis extension for scoping reviews (PRIMA-ScR) to ensure appropriate and relevant methodological information and outcomes are reported (Supplemental file 1).^29^

### Eligibility Criteria

The eligibility criteria were refined using the Population, Intervention, Comparator, Outcome, Timing, and Setting model. These scoping review elements included population (i.e., PCPs, residents, and medical students), interventions of interest (i.e., rural PCP recruitment and retention programs), comparators (i.e., programs reporting concurrent or historic cohorts of participants), outcomes (i.e., programs reporting numerical rural PCP retention after program completion), timing (i.e., studies and programs published from 2013 to 2023 were eligible, regardless of the timing of the intervention), and setting (i.e., retention of PCPs in U.S. rural healthcare areas).

### Search Strategy

In collaboration with a medical librarian, the review team developed a search strategy to locate both published studies and reports to answer the review questions. An initial search was conducted in PubMed to identify keywords and index terms in the titles and abstracts of relevant articles to develop a comprehensive search strategy (Table 1). This search strategy was translated for each database that was searched: PubMed, Embase, and Health Business Elite (see Supplemental file 2 for an example search).

**Table 1:** PubMed Search Terms to Identify Keywords and Index Terms of Relevant Titles and Abstracts to Develop a Comprehensive Search Strategy

**Primary care physician**
(("Primary Health Care"[Mesh] OR "primary care"[tiab] OR "primary health care"[tiab]) AND ("Physicians, Primary Care"[Mesh] OR Physician*[tiab] OR "Medical Doctor"[tiab] OR "doctor of osteopathy"[tiab] OR osteopath*[tiab] OR "Medical Students"[tiab] OR "medical trainee"[tiab] OR "medical trainees"[tiab] OR Residents[tiab] OR Residency[tiab] OR Fellows[tiab] OR Interns[tiab]))
**Recruitment and Retention**
("Personnel Selection"[Mesh] OR "Personnel Staffing and Scheduling"[Mesh] OR "Personnel Turnover"[Mesh] OR "Staff Development"[Mesh] OR "Strikes, Employee"[Mesh] OR "Work Engagement"[Mesh] OR "Workplace"[Mesh] OR "Job Satisfaction"[Mesh] OR "Career Choice"[Mesh] OR "Career Mobility"[Mesh] OR "Motivation"[Mesh] OR "Remuneration"[Mesh] OR "Physician Incentive Plans"[Mesh] OR "Salaries and Fringe Benefits"[Mesh] OR "Education, Medical"[Mesh] OR "Schools, Medical"[Mesh] OR "Socioeconomic Factors"[Mesh] OR "Training Support"[Mesh] OR "Social Responsibility"[Mesh] OR "Community-Institutional Relations"[Mesh] OR "personnel recruitment"[tiab] OR "sustainable rural practice"[tiab] OR "personnel shortage"[tiab] OR "personnel shortages"[tiab] OR "workforce shortage"[tiab] OR "workforce shortages"[tiab] OR "attract and retain"[tiab] OR "recruit and retain"[tiab] OR "recruitment and retention"[tiab] OR "recruiting and retaining"[tiab] OR (under[tiab] AND distrib*[tiab]) OR (improv*[tiab] AND access[tiab) OR (engag*[tiab] AND employ*[tiab]) OR (sustain*[tiab] AND employ*[tiab]) OR (attract*[tiab] AND employ*[tiab]) OR (commit*[tiab] AND employ*[tiab]) OR (workforce[tiab] AND maldistribut*[tiab]) OR (interest*[tiab] AND employ*[tiab]) OR (encourag*[tiab] AND employ*[tiab]) OR (work*[tiab] AND satisfaction*[tiab]) OR (career[tiab] AND advance*[tiab]) OR "unmet need"[tiab] OR "workforce need"[tiab] OR "workforce needs"[tiab] OR "recruitment strategy"[tiab] OR "recruitment strategies"[tiab] OR (retention[tiab] AND strateg*[tiab]) OR "career development"[tiab] OR (plan*[tiab] AND workforce[tiab]) OR Recruit[tiab] OR Recruitment[tiab] OR Retain*[tiab] OR "J-1 visa waiver"[tiab] OR "Area Health Education Center"[tiab] OR "Loan Repayment Programs"[tiab] OR "Relocation Allowance"[tiab] OR "Physician supply"[tiab] OR "Physician Shortage"[tiab] OR "vacancy rate"[tiab] OR "vacancy rates"[tiab] OR "duration of service"[tiab] OR "financial incentive"[tiab] OR "financial incentives"[tiab] OR "financial inducement"[tiab] OR "financial inducements"[tiab] OR "monetary incentive"[tiab] OR "monetary incentives"[tiab] OR (non-financial[tiab] AND inducement*[tiab]) OR "non-monetary incentive"[tiab] OR "non-monetary incentives"[tiab] OR (incentiv*[tiab] AND measure*[tiab]) OR (incentiv*[tiab] AND polic*[tiab]) OR "faculty development"[tiab] OR "professional development" OR "rural exposure"[tiab] OR "rural learning experiences"[tiab] OR "rural scholarship"[tiab] OR "educational grant"[tiab] OR "educational grants"[tiab] OR "community participation"[tiab] OR "social accountability"[tiab])
**Rural Areas**
("Rural Population"[Mesh] OR "Rural Health Services"[Mesh] OR "Rural Health"[Mesh] OR "Hospitals, Rural"[Mesh] OR "Medically Underserved Area"[Mesh] OR "rural area"[tiab] OR "rural areas"[tiab] OR "rural community"[tiab] OR "rural communities"[tiab] OR "rural location"[tiab] OR "rural locations"[tiab] OR "rural practice"[tiab] OR "rural practices"[tiab] OR "remote area"[tiab] OR "remote areas"[tiab] OR "remote community"[tiab] OR "remote communities"[tiab] OR "remote location"[tiab] OR "remote locations"[tiab] OR "remote practice"[tiab] OR "remote practices"[tiab] OR "underserved area"[tiab] OR "underserved areas"[tiab] OR "underserved location"[tiab] OR "underserved locations"[tiab] OR "underserved community"[tiab] OR "underserved communities"[tiab] OR "geographically isolated area"[tiab] OR "geographically isolated areas"[tiab] OR "geographically isolated community"[tiab] OR "geographically isolated communities"[tiab] OR "island community"[tiab] OR "island communities"[tiab] OR "islands community"[tiab] OR "islands communities"[tiab] OR "remote island community"[tiab] OR "remote island communities"[tiab] OR "remote islands community"[tiab] OR "remote islands communities"[tiab] OR "poorly served area"[tiab] OR "poorly served areas"[tiab] OR "poorly served community"[tiab] OR "poorly served communities"[tiab] OR "underserviced area"[tiab] OR "underserviced areas"[tiab] OR "rural and remote area"[tiab] OR "rural and remote areas"[tiab] OR "Health Profession Shortage Area"[tiab] OR "HPSA"[tiab] OR "Medically Underserved Area"[tiab] OR "MUA"[tiab] OR (rural[tiab] AND (physician*[tiab] OR doctor[tiab] OR hospital*[tiab] OR communit*[tiab] OR medicine[tiab])))

Additionally, a thorough search of the gray literature was conducted using the Rural Health Information (RHI) Hub, which serves as a state and national funding opportunity repository.^30^ From the RHI, rural funding search terms included *Awards (Monetary), Educational Opportunities and Fellowships*,* Incentives*,* Loan Repayment Programs (LRPs)*, *Loans*, and *Scholarships*. Websites of relevant programs identified through the RHI hub were searched for any type of retention statistics or reporting. Additionally, we searched the Rural Medical Training Collaborative (RMTC), which “maintains a database of rural programs in both undergraduate and graduate medical education, including demographics and outcomes.”^31^ The RMTC provides a map of national rural education programs, as well as a curated list of select programs. Using these two resources, we searched rural education programs’ websites for retention statistics or reporting (programs previously identified via our exploration of the RHI hub were not searched again). Finally, a Google search was conducted to identify any additional rural PCP programs that were not found through the RHI hub or RMTC. Terms searched included *Department of Veterans Affairs-sponsored incentive programs for rural practitioners*,* rural* PCP, or *family medicine incentive programs*. Pages were searched until saturation was reached, operationalized as three consecutive pages that no longer provided new relevant information.

### Study Selection

Overall, the search strategy across the 3 databases yielded 2171 studies, which were imported into Covidence, a systematic-review management software,^32^ for title and abstract review. Each title and abstract was reviewed by two independent screeners (KA, MS, KB, or LW), and conflicts were resolved by a third reviewer (HT). This review included all major primary and secondary study designs, including randomized and nonrandomized controlled trials, prospective and retrospective cohort studies, case–control studies, cross-sectional studies, case studies/reports, and evaluation studies. It also included other systematic and scoping reviews and meta-analyses. Commentaries, narratives, perspectives, editorials, and studies for which the full text was unavailable were excluded. Articles selected for full-text review were independently evaluated based on the inclusion criteria by two reviewers (KA, KB, MS, LW, or APR), with conflicts resolved by a third reviewer (HT). Studies were required to discuss U.S.-based programs in English and be published between 2013 and 2023 to optimize the possibility that programmatic results were reported versus just a description of a new program. Furthermore, the inclusion criteria for full-text review stipulated those articles needed to:

1. Focus on rural PCP workforce development;
2. Describe a specific rural PCP recruitment or retention program; and
3. Discuss outcomes related to the number of PCPs practicing in rural areas after the rural recruitment or retention program ended.

Articles were included that met all three inclusion criteria. Conversely, articles were excluded that did not:

1. Appear in English;
2. Discuss recruitment or retention programs in the U.S.;
3. Focus on rural PCP recruitment or retention;
4. Explicitly say or define rural area; and/or
5. Report the intersection of rural and PCP retention outcomes after program completion.

### Data Collection Process and Data Items

Among the peer-reviewed articles meeting inclusion criteria, data from each article were extracted by two coders (LW, MS, or APR) and reviewed by the first author (KA) for consensus. Data were then downloaded from Covidence into a spreadsheet. The gray literature review yielded additional programs that met inclusion criteria. Data from these programs were extracted and collated into the literature search spreadsheet.

Data items collected for each program included elements from:

- *Population:* PCPs, residents, and medical students,
- *Concept:* recruitment or retention programs specifically focused on rural PCP workforce development,
- *Context:* rural area, and
- *Expected outcomes:* outcomes related to the number of PCPs practicing in rural areas after the rural recruitment or retention program ended.

### Data Synthesis

Since programs did not report retention results systematically, descriptive data from the programs were analyzed thematically (see Table 2). Data from each program were extracted, including program name; year program started; program duration; whether the program was focused on recruitment, retention, or both; and reported retention results. In alignment with the WHO Global Policy Recommendation, programs were categorized as education, regulation, financial incentives, and personal and professional support.^33^ Next, programs were further categorized by subtype, which included state loan repayment program; residency, clerkship, or clinical rotation; and scholarship with service time. Furthermore, the career stage (e.g., undergraduate students, medical students, or residency) was categorized for each program.

**Table 2:** Description of Programs by Category

Program category	Subtype	Program name	Recruit or retain	Data years included	Sample size	HPSA score and rural status	Results
Education	Residency, clerkship, or clinical rotation	The Family Medicine Residency of Western Montana	Recruit	2013–2024	*n* = 89	14, partially rural	68% of graduates practice in rural/underserved locations
Education	Residency, clerkship, or clinical rotation	Cascades East Family Medicine Residency	Recruit	1994–2009	*n* = 62	15, rural	50% of all the graduates remain in rural settings
Education	Residency, clerkship, or clinical rotation	Rural Family Medicine Residency Program	Recruit	2021–2024	*n* = 4	20, non-rural	50% of the first class chose to stay in rural communities in which they trained
Education	Residency, clerkship, or clinical rotation	Family Medicine Residency of Idaho – Caldwell program	Recruit	1995–2024	*n* = 54*	13, partially rural	88% are serving in rural areas
Education	Residency, clerkship, or clinical rotation	The Teaching Health Center Graduate Medical Education program	Recruit	2014–2017	*n* = 533	N/A, in multiple counties	12% of primary care physicians (53 of 445) were practicing in rural areas 51% were practicing in an HPSA/MUA
Education	Residency, clerkship, or clinical rotation	Marshall University Family Medicine Residency Rural Track	Recruit	1994–2006	*n* = 12	15, non-rural	83.3% (n = 10) of the Rural Track graduates practiced in a rural area
Education	Residency, clerkship, or clinical rotation	Iowa Family Medicine Training Network	Recruit	1977–2014	*n* = 1645	16, non-rural	Of the graduates practicing in Iowa, 47.3% were in rural communities
Education	Residency, clerkship, or clinical rotation	Rural and rural training track residency programs	Recruit	2008–2018	*n* = 682	N/A, multiple counties	Rural track graduates spend 52% of practice years in rural areas compared to non-rural track graduates (18% of practice years in rural locations)
Education	Residency, clerkship, or clinical rotation	John Peter Smith Family Medicine Residence, Advanced Rural/Global Medical Services track	Recruit	2007–2016	*n* = 9	Non-HPSA, non-rural	33.33% are practicing in HPSA 33.33% are practicing in a "Rural" area
Education	Rural education program	Rural Medical Scholars Program	Recruit	1996–2013	*n* = 126	20, non-rural	Of the 165 rural Alabama students who have entered the program since its founding, more than 60 percent have completed their training and are practicing as primary care physicians in Alabama rural communities
Education	Rural education program	Physician Shortage Area Program	Recruit	1978–2002	*n* = > 300*	10, non-rural	Graduates from 2 different cohorts (1978–1991, and 1992–2002) were 8.5 to 9.9 times more likely to practice rural family medicine than their non-rural track classmates Approximately 70% of graduates have remained in rural family practice in the same area for at least 20 to 25 years after first located in practice, and an additional 10% had previously moved to another rural area
Education	Rural education program	Rockford Rural Medical Education Program	Recruit	1997–2007	*n* = 160	16, non-rural	47% of graduates are rural primary care physicians 43% of graduates are rural family medicine physicians
Education	Rural education program	Rural and Urban Scholars Pathway	Recruit	2013–2023	*n* = 14	19, rural	43% (n = 6) are practicing primary care in rural locations
Financial Incentive	Other: scholarship with service commitment + academic and professional enrichment	Mississippi Rural Physicians Scholarship Program	Recruit	2007–2022	*n* = 24	20, non-rural	87.5% are still practicing in rural towns in Mississippi after completing their service time commitment
Regulation	State loan repayment program	Michigan State Loan Repayment Program	Recruit	2017–2019	*n* = 3	N/A, program applies statewide	3 out of 3 MD's are still working at their MSLRP approved practice site
Regulation	State loan repayment program	Vermont AHEC Educational loan repayment program	Recruit	2015–2018	*n* = 126	NA, program applies statewide	There were 99 awardees from the 2015–2018 cohort working in a rural and/or federally designated worksite in 2019
Regulation	Scholarship with service time	West Virginia University Institute for Community and Rural Health service award program	Recruit	2011–2020	*n* = 4	18, non-rural	Of the medical recipients that completed the program, 2 fulfilled requirements and 2 repaid loans. Of these, 2 are working in rural areas

*Estimated n size based on description in manuscript or text

#### HPSAs

To visualize the geographic distribution of programs relative to rural primary care HPSAs, we created a map displaying program locations and retention outcomes over rural primary care HPSAs using ArcGIS Pro 3.2.0.^34^ Data from the Health Resources and Services Administration were used to identify all primary care HPSAs in rural counties. Primary care HPSA scores are evaluated on population-to-provider ratio, percent of individuals below the federal poverty level, infant health index, and average travel time to nearest source of care. Scores range from 1 to 25, with higher scores indicating greater priority. Programs housed within institutions were plotted at the central point of the county where the institution’s main campus is located to display the proportion of PCPs. State-level programs were plotted at the central point in the state to display the proportion of PCPs retained in rural practice. Articles reporting retention results aggregated across institutions were not displayed on the map.

#### Retention Spectrum Display

A spectrum display was used to compare qualitative characteristics of the included different programs. Reported retention rates were compared between programs designated as (1) Residency, Clerkship, or Clinical Rotation; (2) Rural Education Program; (3) Scholarship and Service; or (4) State Loan Repayment Program. Programs were categorized as those with retention rates under 50%, those between 50 and 75%, and those ranging from 75% to 100% and ordered using a combination of Program Category (Education, Regulation, or Financial Incentive) and retention rate.

## RESULTS

**Figure 1:**
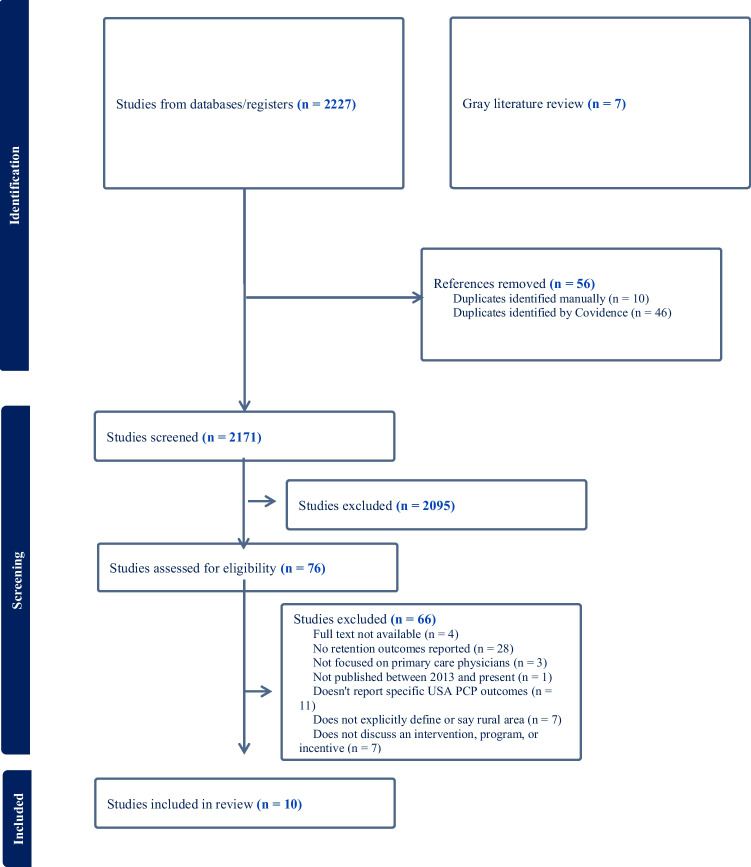
PRISMA flow diagram of rural primary care recruitment and retention program review process.

The search strategy yielded 2227 articles from PubMed, Embase, and Health Business Elite (see Fig. 1). Fifty-six articles were identified as duplicates and removed. After reviewing the titles and abstracts of 2171 articles, we excluded 2,095 articles, and 76 articles’ full texts were reviewed. Of these, only ten met the criteria for inclusion. From the gray literature review, 7 nonduplicate programs were eligible for inclusion, totaling 17 unique programs included in the present review (Table 2; see Supplemental 3 for a complete list of programs and data extracted).

One program focused on both recruitment and retention, while the remaining 16 focused only on recruitment. Although all programs had a primary care workforce development focus, Table 3 shows which primary care areas were specified across programs and where most specified family medicine (*n* = 12), followed by the inclusion of PCPs in general (*n* = 9). Additionally, programs targeted different stages of the student-to-rural physician pipeline, with most implemented during residency (*n* = 10) followed by medical school (*n* = 6; see Table 4).

**Table 3:** Recruitment and Retention Programs Preferred Primary Care Provider Focus Areas

Primary care provider focus	Frequency
Primary care providers in general	9
Family medicine	12
General internal medicine	3
General pediatrics	3
Obsterics/gynecology	2

Most programs specified multiple primary care focus areas; therefore, the total count exceeds the total number of individual programs

**Table 4:** Career Stage Primary Care Physician Recruitment and Retention Programs Target

Career stage	Frequency
Undergraduate	2
Medical school	6
Residency	10
Early career	1
Practicing physician	2

Some programs were implemented at multiple career stages; therefore, the total count exceeds the total number of individual programs

Retention outcomes should be cautiously interpreted since sample sizes and the years between program completion and current practice site varied greatly across programs. Retention outcomes varied across program types, with state loan repayment programs (*n* = 2) exhibiting the highest retention rates (75–100%; *n* size for each study was 3 and 126, respectively), followed by scholarship services (*n* = 2) with retention rates from 50% (study participants *n* = 4) to 88% (study participants *n* = 24).

**Figure 2:**
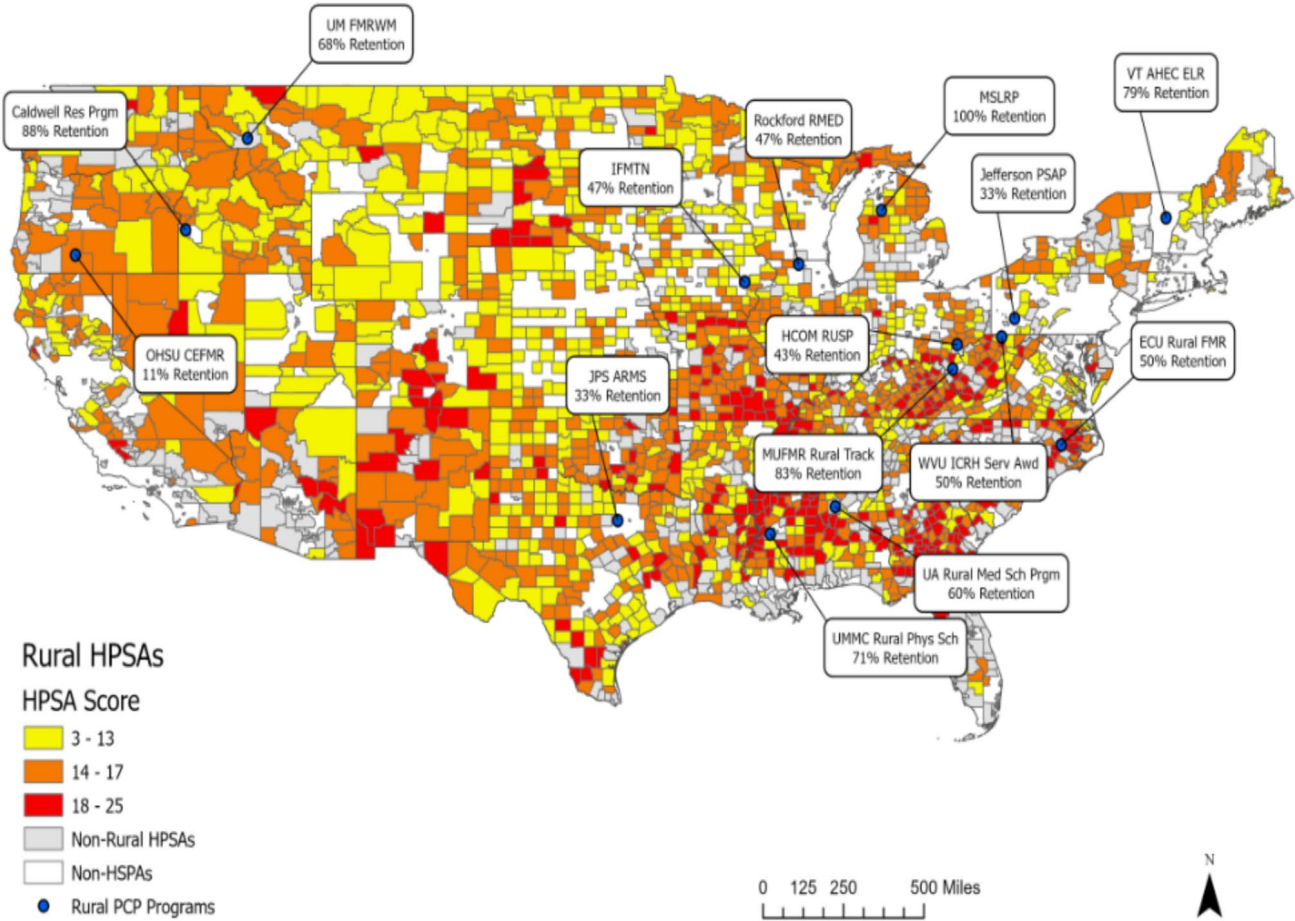
U.S. map with HPSA scores and retention outcomes reported by 15 of the 17 rural primary care provider recruitment and retention programs

Figure 2 is a map that displays the geographic locations and retention outcomes reported by 15 of the 17 programs. Articles by Strasser et al.^32^ and Meyers et al.^33^ report retention outcomes aggregated across multiple institutions, so they were not displayed on the map. Note that, apart from the Michigan State Loan Repayment Program and the Vermont Advanced Health Education Center Educational Loan Repayment Program, the location of the points corresponds to locations of the main campuses of institutions, but residents may provide patient care in nearby counties. Of the 13 programs housed within institutions, only 2 have a main campus in rural counties that are primary care HPSAs: Cascades East Family Medicine Residency at Oregon Health & Sciences University in Klamath County, Oregon (a low-income HPSA with a score of 15), and Rural and Urban Scholars Pathway at Heritage College of Osteopathic Medicine, Ohio University in Athens County, Ohio (a low-income HPSA with a score of 19). Nine programs are in non-rural counties considered primary care HPSAs, and two are in counties without HPSA designation. With respect to the state-level loan repayment programs, Michigan has 49 rural primary care geographic and population HPSAs, whereas the state of Vermont has only 1 rural primary care low-income HPSA.

**Figure 3:**
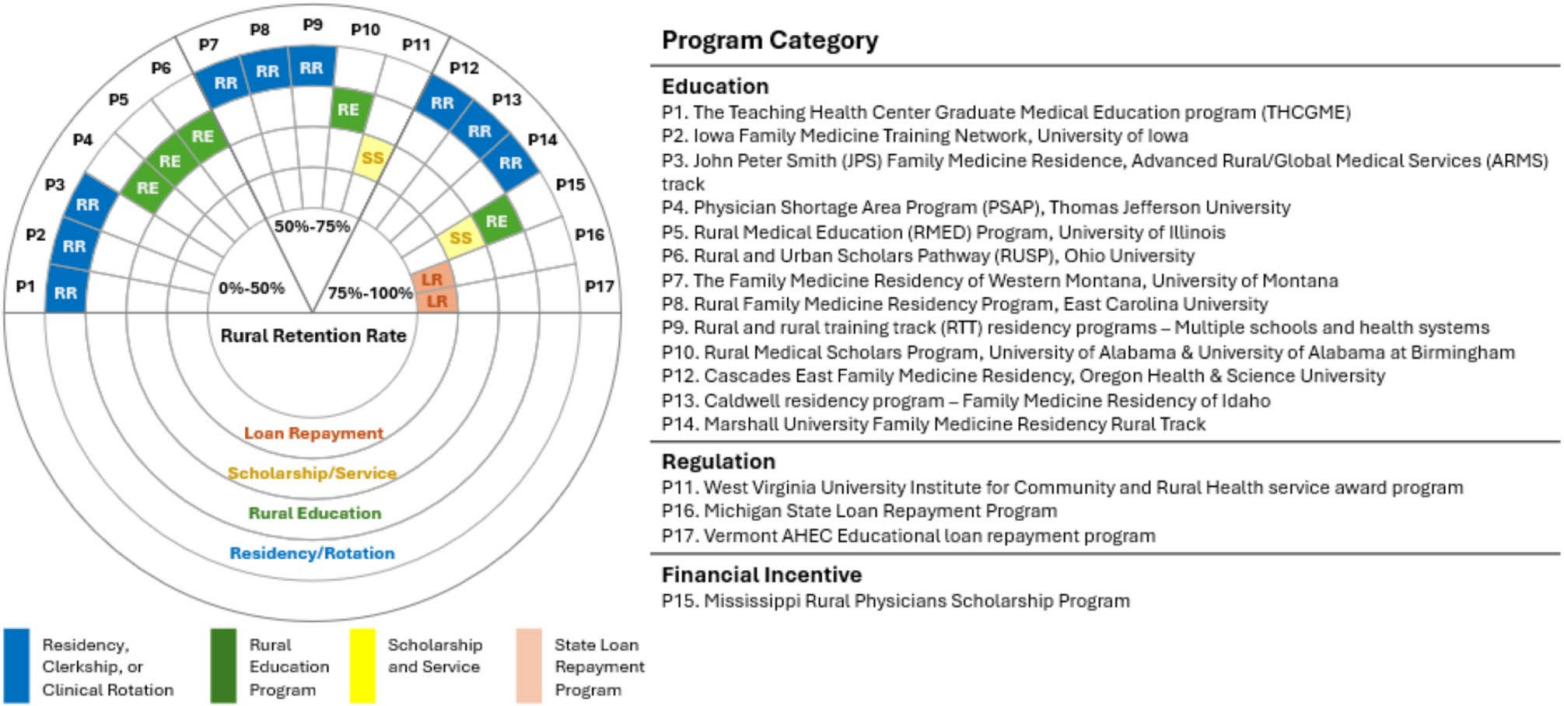
Spectrum display of the qualitative features of the 17 programs organized by the unweighted proportion of participating primary care providers who were retained within rural care

The spectrum display in Fig. 3 portrays the qualitative features of the 17 programs organized by the unweighted proportion of participating PCPs who were retained within rural care. Among those studied, programs offering State Loan Repayment Programs had the highest retention (n = 2, 75–100%), followed by Scholarship Services (n = 2, 50–100%). Programs characterized as Residency, Clerkship, or Clinical Rotation were evenly distributed across retention categories, while Rural Education Programs had proportionately lower average retention than the others evaluated.

## DISCUSSION

This scoping review examined the effectiveness of rural PCP recruitment and retention programs in the U.S. Despite significant investments, many programs do not report retention outcomes, limiting the ability to assess their long-term success. When physicians leave shortage areas, continuity of care suffers and institutions lose the resources they invested in physicians’ development.^26,35^ Therefore, it is critical to understand what characteristics are most successful to ensure recruitment and retention programs contain these critical elements to increase rural retention.

Findings from related workforce studies echo the patterns found in this scoping review. Ceric et al. identified that, in addition to financial incentives, people who stay in their relocated area share unique personal, internal motivators to relocate and identify clear opportunities for career progression in their decision to move.^36^ In this review, both residency, clerkship, or clinical rotation programs and rural education programs showed large variability in retention outcomes, with incongruous methods for measuring and reporting program retention and overall success. The results highlight the need for more standardized and systematic reporting of retention outcomes to better understand which programs are most effective.

A key overarching finding is the pervasive lack of systematically collected and publicly available retention outcome data across rural PCP recruitment and retention programs. This gap is itself a meaningful result; without consistent tracking of long-term practice outcomes or shared definitions of “rural” and “retention,” comparisons across programs are not feasible, and true effectiveness remains unclear. Strengthening rural workforce capacity will require coordinated expectations for retention metrics and transparent reporting at institutional, state, and federal levels. Without these foundational data, decision-makers cannot determine which models warrant continued investment or which program components most directly support long-term rural PCP retention.

### Strengths, Limitations, and Future Directions

This review has several strengths. At present, this is the largest and most comprehensive review of the existing literature comparing strategies for recruiting and retaining PCPs in rural communities. The broad range of sources and rigorous search approach enabled the identification of key themes shaping program success.

However, some limitations impact the interpretation of these findings. First, the program heterogeneity presented a significant challenge. Programs varied widely in terms of structure, duration, historical longevity (i.e., 2 cohorts of participants vs 20 cohorts of participants), and targeted career stages, making direct comparisons difficult. Sample sizes and follow-up periods also varied considerably, underscoring the need for standardized reporting criteria.

Second, the limited availability of longitudinal data limited our ability to assess whether initial recruitment translated into sustained rural practice beyond mandatory service commitments. Long-term follow-up is essential for understanding true workforce impact.

Third, the quality and consistency of retention data reporting were variable. Some programs provided detailed data, while others reported only partial information, such as how many were currently practicing in rural areas but did not mention physicians’ specialty areas or how many years individuals stayed in rural areas after the service time commitment was completed. Additionally, many programs excluded from analysis reported physicians’ intent to practice in rural areas; however, this is not a proxy variable for retention. To advance the field, programs should at minimum report the number of PCPs (not just physicians in general) that (a) participated in the program, (b) completed the program, (c) are practicing in the original rural placement, (d) are practicing in a rural area within the program’s state, (e) are practicing in a rural area outside the state; and (f) duration of rural practice following program completion. Standardized metrics and reporting guidelines are needed to ensure that retention outcomes are consistently and accurately reported.

Several barriers impede consistent reporting, including fragmented data systems, varying definitions of key terms, limited capacity for long-term tracking, and a lack of mandated reporting requirements. Addressing these barriers will require shared reporting standards and stronger infrastructure for centralized data collection at state and federal levels, potentially through HRSA, state workforce offices, and licensure boards. Enhanced data systems would improve transparency, support comparative effectiveness research, and ensure that program investments align with sustainable rural PCP retention.

Future research should also investigate the role of other elements that influence recruitment and retention. As supported by the broader workforce literature, recruitment and retention are influenced not only by financial and educational support but also by organizational, interpersonal, and cultural factors. These elements shape clinicians’ daily experiences and include workplace climate, community integration, professional identity fit, job satisfaction, and workload. Future rural PCP workforce efforts would benefit from systematically evaluating these contextual and cultural dimensions as they may meaningfully influence whether clinicians feel committed to and integrated within their rural practice environment.

A critical next step involves examining predictors of intent to leave, a well-established antecedent to actual turnover.^36^ Understanding the time and space between intent to leave and the eventual decision to exit rural practice could inform earlier, more targeted interventions. For example, Chandra et al. developed a “morale index” capturing different elements of the work environment which predicted intent to leave among hospitalists.^37^ Applying similar measures to rural PCPs may help identify early warning signs and modifiable contributors to departure from rural clinics. Integrating predictive tools with program evaluation could deepen understanding of the mechanisms that drive retention and provide actionable insights for policymakers and institutional leaders.

## CONCLUSION

Some rural recruitment and retention programs, especially those involving financial incentives and educational support, could show promise in abating the current rural PCP shortage by influencing PCPs to remain in rural practice post program completion. However, significant gaps remain. Further research is needed to establish robust evidence on program effectiveness, identify best practices, and address ongoing challenges in rural healthcare workforce development. Specifically, standardized reporting, long-term studies, comparative research, and comprehensive program design are crucial for mitigating PCP shortages and improving healthcare access in underserved rural communities across the U.S.

## Supplementary Information

Below is the link to the electronic supplementary material.

Supplementary file1 (PDF 636 KB)

Supplementary file2 (DOCX 29.1 KB)

Supplementary file3 (XLSX 26.6 KB)

## Data Availability

Data can be found in Supplementary File 3.
