# Supplementary material for: Rural Practice Made Attractive: A Scoping Review of Rural Primary Care Physician Recruitment and Retention Incentives
Source: J Gen Intern Med. 2026 Jan 26;41(5):1367–78. doi: 10.1007/s11606-026-10218-8 (PMC13083479; doi:10.1007/s11606-026-10218-8)
Supplement: Supplementary file 2 — Supplementary file2 (DOCX 29.1 KB) [file 11606_2026_10218_MOESM2_ESM.docx]

Rural Practice Made Attractive: A Scoping Review of Rural Primary Care Physician Recruitment and Retention Incentives

Kelley Arredondo, PhD,^1,2,3,4^ Katherine Bay, PhD,^1^ Laura Witte, DrPH,^5^ Hilary Touchett, PhD, RN,^1,2,4^ Mandi Sonnenfeld, PhD, MOT,^1,2^ Alexander Paterson-Roberts, BBA,^6^ Matthew Vincenti, PhD,^3,7^ Bradley V. Watts, MD, MPH^3,8^

1. Houston VA HSR&D Center for Innovations in Quality, Effectiveness, and Safety, Michael E. DeBakey VA Medical Center
2. Department of Medicine, Baylor College of Medicine, Houston, TX
3. VHA Office of Rural Health’s Veterans Resource Center in White River Junction, VT
4. VA South Central Mental Illness Research, Education, Clinical Center, a Virtual Center
5. Veterans Affairs, South Texas Veterans Health Care System, San Antonio, TX
6. Department of Psychology, University of Houston, Houston, TX
7. Department of Medicine, Dartmouth Geisel School of Medicine, New Hampshire, VT
8. Department of Psychiatry, Larner School of Medicine at the University of Vermont, Burlington, VT

**Supplemental file 2.** Search Strategy

**Embase on Ovid platform**

Embase <1974 to 2023 April 13>

1 primary medical care/

2 "primary care".ab,ti.

3 "primary health care".ab,ti.

4 "primary medical care".ab,ti.

5 1 or 2 or 3 or 4

6 physician/

7 ("medical trainee" or "medical trainees").ab,ti.

8 osteopathic physician/

9 residency education/

10 medical education/

11 resident/

12 "physician*".ab,ti. 648719

13 medical.ab,ti. 2109632

14 doctor.ab,ti. 85198

15 "osteopath*".ab,ti. 7932

16 "residen*".ab,ti. 399990

17 "fellow*".ab,ti. 55782

18 "intern*".ab,ti. 1571086

19 6 or 8 or 9 or 10 or 11 or 12 or 13 or 14 or 15 or 16 or 17 or 18 4499106

20 5 and 19 120236

21 personnel management/ 59544

22 "organization and management"/ or hospital management/ 457533

23 labor strike/ 20

24 work engagement/ 2517

25 workplace/ 53649

26 job satisfaction/ 35130

27 decision making/ 279383

28 career planning/ or career mobility/ 14237

29 remuneration/ or "salary and fringe benefit"/ 14729

30 incentive/ or economic incentive/ or social incentive/ 4011

31 medical education/ 248693

32 medical school/ 69557

33 socioeconomics/ 159144

34 social responsibility/ 3202

35 community participation/ or community integration/ or community/ or community program/ 95582

36 "personnel recruitment".ab,ti. 43

37 "sustainable rural practice".ab,ti. 2

38 "personnel shortage".ab,ti. 121

39 "personnel shortages".ab,ti. 165

40 "workforce shortage".ab,ti. 620

41 "workforce shortages".ab,ti. 916

42 "attract and retain".ab,ti. 544

43 "recruit and retain".ab,ti. 1096

44 "recruitment and retention".ab,ti. 7407

45 "recruiting and retaining".ab,ti. 1015

46 (under and distrib*).ab,ti. 151436

47 (improv* and access).ab,ti. 154727

48 (engag* and employ*).ab,ti. 20629

49 (sustain* and employ*).ab,ti. 22563

50 (attract* and employ*).ab,ti. 13543

51 (commit* and employ*).ab,ti. 15434

52 (workforce and maldistribut*).ab,ti. 241

53 (interest* and employ*).ab,ti. 53586

54 (encourag* and employ*).ab,ti. 13429

55 (work* and satisf*).ab,ti. 78653

56 (career and advance*).ab,ti. 5162

57 "unmet need".ab,ti. 22436

58 "workforce need".ab,ti. 50

59 "workforce needs".ab,ti. 580

60 "recruitment strategy".ab,ti. 1278

61 "recruitment strategies".ab,ti. 2813

62 (retent* and strateg*).ab,ti. 21767

63 "career development".ab,ti. 4005

64 (plan* and workforce).ab,ti. 6690

65 "recruit*".ab,ti. 715042

66 "retain*".ab,ti. 289689

67 "J-1 visa waiver".ab,ti. 10

68 "Area Health Education Center".ab,ti. 149

69 "Loan Repayment Programs".ab,ti. 37

70 "Relocation Allowance".ab,ti. 1

71 "Physician supply".ab,ti. 645

72 "Physician Shortage".ab,ti. 454

73 "Relocation Allowance".ab,ti. 1

74 "Physician supply".ab,ti. 645

75 "Physician Shortage".ab,ti. 454

76 "Physician Shortages".ab,ti. 319

77 "vacancy rate".ab,ti. 125

78 "vacancy rates".ab,ti. 170

79 "duration of service".ab,ti. 241

80 "financial incentive".ab,ti. 1416

81 "financial incentives".ab,ti. 5855

82 "financial inducement".ab,ti. 9

83 "financial inducements".ab,ti. 34

84 "monetary incentive".ab,ti. 1313

85 "monetary incentives".ab,ti. 973

86 (non-financial and inducement*).ab,ti. 2

87 "non-monetary incentive".ab,ti. 16

88 "non-monetary incentives".ab,ti. 107

89 (incentiv* and measure*).ab,ti. 9803

90 (incentiv* and polic*).ab,ti. 8488

91 "faculty development".ab,ti. 3845

92 "professional development".ab,ti. 15435

93 "rural exposure".ab,ti. 80

94 "rural learning experiences".ab,ti. 4

95 "rural scholarship".ab,ti. 4

96 "educational grant".ab,ti. 463

97 "educational grants".ab,ti. 162

98 "community participation".ab,ti. 4210

99 "social accountability".ab,ti. 478

100 21 or 22 or 23 or 24 or 25 or 26 or 27 or 28 or 29 or 30 or 31 or 32 or 33 or 34 or 35 or 36 or 37 or 38 or 39 or 40 or 41 or 42 or 43 or 44 or 45 or 46 or 47 or 48 or 49 or 50 or 51 or 52 or 53 or 54 or 55 or 56 or 57 or 58 or 59 or 60 or 61 or 62 or 63 or 64 or 65 or 66 or 67 or 68 or 69 or 70 or 71 or 72 or 73 or 74 or 75 or 76 or 77 or 78 or 79 or 80 or 81 or 82 or 83 or 84 or 85 or 86 or 87 or 88 or 89 or 90 or 91 or 92 or 93 or 94 or 95 or 96 or 97 or 98 or 99 2779847

101 rural population/ 55680

102 rural health care/ 14577

103 rural health/ 2015

104 rural hospital/ 1243

105 medically underserved/ 1669

106 "rural area".ab,ti. 15278

107 "rural areas".ab,ti. 46478

108 "rural community".ab,ti. 7248

109 "rural communities".ab,ti. 10861

110 "rural location".ab,ti. 1484

111 "rural locations".ab,ti. 1300

112 "rural practice".ab,ti. 1477

113 "rural practices".ab,ti. 334

114 "remote area".ab,ti. 1308

115 "remote areas".ab,ti. 7177

116 "remote community".ab,ti. 358

117 "remote communities".ab,ti. 1779

118 "remote location".ab,ti. 780

119 "remote locations".ab,ti. 2011

120 "remote practice".ab,ti. 104

121 "remote practices".ab,ti. 21

122 "underserved area".ab,ti. 373

123 "underserved areas".ab,ti. 2064

124 "underserved location".ab,ti. 5

125 "underserved locations".ab,ti. 34

126 "underserved community".ab,ti. 459

127 "underserved communities".ab,ti. 2060

128 "geographically isolated area".ab,ti. 25

129 "geographically isolated areas".ab,ti. 75

130 "island community".ab,ti. 177

131 "island communities".ab,ti. 233

132 "islands community".ab,ti. 11

133 "islands communities".ab,ti. 9

134 "remote island community".ab,ti. 10

135 "remote island communities".ab,ti. 8

136 "remote islands community".ab,ti. 0

137 "remote islands communities".ab,ti. 0

138 "poorly served area".ab,ti. 0

139 "poorly served areas".ab,ti. 4

140 "poorly served community".ab,ti. 1

141 "poorly served communities".ab,ti. 0

142 "underserviced area".ab,ti. 11

143 "underserviced areas".ab,ti. 65

144 "rural and remote area".ab,ti. 87

145 "rural and remote areas".ab,ti. 1412

146 "Health Profession Shortage Area".ab,ti. 2

147 "HPSA".ab,ti. 508

148 "Medically Underserved Area".ab,ti. 148

149 "MUA".ab,ti. 9856

150 (hospital or hospitals).ab,ti. 2178405

151 physician*.ab,ti. 648719

152 doctor*.ab,ti. 214335

153 medicine.ab,ti. 858280

154 communit*.ab,ti. 897318

155 150 or 151 or 152 or 153 or 154 4211314

156 rural.ab,ti. 206604

157 155 and 156 91815

158 101 or 102 or 103 or 104 or 105 or 106 or 107 or 108 or 109 or 110 or 111 or 112 or 113 or 114 or 115 or 116 or 117 or 118 or 119 or 120 or 121 or 122 or 123 or 124 or 125 or 126 or 127 or 128 or 129 or 130 or 131 or 132 or 133 or 134 or 135 or 136 or 137 or 138 or 139 or 140 or 141 or 142 or 143 or 144 or 145 or 146 or 147 or 148 or 149 or 157 188988

159 20 and 100 and 158 3207

Last 10 years 1899 results
